# New Insights into Evolution of the ABC Transporter Family in *Mesostigma viride*, a Unicellular Charophyte Algae

**DOI:** 10.3390/cimb44040112

**Published:** 2022-04-11

**Authors:** Xiaoping Gong, Shanhong Wang

**Affiliations:** National Engineering Research Center of Crop Molecular Breeding, Institute of Crop Sciences, Chinese Academy of Agricultural Sciences, Beijing 100081, China; wangshanhong@caas.cn

**Keywords:** ABC transporter, green plant, phylogeny, *Mesostigma viride*, evolution

## Abstract

ATP-binding cassette (ABC) transporters play an important role in driving the exchange of multiple molecules across cell membranes. The plant ABC transporter family is among the largest protein families, and recent progress has advanced our understanding of ABC classification. However, the ancestral form and deep origin of plant ABCs remain elusive. In this study, we identified 59 ABC transporters in *Mesostigma viride*, a unicellular charophyte algae that represents the earliest diverging lineage of streptophytes, and 1034 ABCs in genomes representing a broad taxonomic sampling from distantly related plant evolutionary lineages, including chlorophytes, charophytes, bryophytes, lycophytes, gymnosperms, basal angiosperms, monocots, and eudicots. We classified the plant ABC transporters by comprehensive phylogenetic analysis of each subfamily. Our analysis revealed the ancestral type of ABC proteins as well as duplication and gene loss during plant evolution, contributing to our understanding of the functional conservation and diversity of this family. In summary, this study provides new insight into the origin and evolution of plant ABC transporters.

## 1. Introduction

The ATP-binding cassette (ABC) transporter is an ancient and large family of transmembrane transport proteins [1,2,3], which are present in all cellular organisms and participate in the transport of a variety of substances [4,5,6,7,8,9,10]. Based on their structural organization, ABC proteins are mainly divided into full- and half-transporters. Full transporters are composed of two transmembrane domains (TMD) and two nucleotide-binding domains (NBD). Half transporters are composed of one TMD and one NBD [11,12].

In plants, ABC transporters are one of the largest protein families and include eight subfamilies (ABCA-G and ABCI) [11,13]. During evolution, plant ABC transporter genes have undergone multiplication and functional diversification [14,15,16]. The high number and complexity of ABC proteins requires comprehensive phylogenetic study to resolve their evolution and function. Several studies have reported the classification of ABC transporters in plants [17,18,19]. However, an investigation of ABCs in unicellular charophyte algae is missing and is crucial to understand the ancestral form and the original function of the plant ABC family.

All green plants can be divided into two groups: Streptophyta and chlorophyte green algae. Streptophyta are further divided into charophyte green algae and all land plants, which evolved from unicellular charophyte algae predecessors [20,21]. Currently, a total of six charophyte green algae genome have been reported [22,23,24,25,26]. *Mesostigma viride* is the only unicellular charophyte algae that was recently genome sequenced and is representative of the earliest diverging lineage of streptophytes [24].

In this study, we searched for ABC transporters in M. viride and evolutionarily representative genomes, including Chlamydomonas reinhardtii, Volvox carteri, Klebsormidium flaccidum, Physcomitrella patens, Marchantia polymorpha, Selaginella moellendorffii, Picea abies, Amborella trichopoda, Oryza sativa, and the model plant Arabidopsis thaliana. We performed comprehensive phylogenetic analysis and classified the ABC subfamilies.

## 2. Materials and Methods

### 2.1. Sequence Retrieval 

We performed BLASTP and TBLASTN searches using well-studied *Arabidopsis* ABC proteins [11,27] from each subfamily as queries to identify the plant ABCs (e-value  <  e-10) from the 11 selected plants: *M. viride* (https://phycocosm.jgi.doe.gov/Mesvir1/Mesvir1.home.html, accessed on 1 May 2021), *C. reinhardtii* (http://plants.ensembl.org/Chlamydomonas_reinhardtii/Info/Index, accessed on 7 May 2021), *V. carteri* (https://bioinformatics.psb.ugent.be/plaza/versions/plaza_v2_5/download/index, accessed on 6 May 2021), *K. flaccidum* (http://www.plantmorphogenesis.bio.titech.ac.jp/~algae_genome_project/klebsormidium/kf_download.htm, accessed on 8 May 2021), *M. polymorpha* (http://marchantia.info/download/, accessed on 15 May 2021), *P. patens* (https://plants.ensembl.org/Physcomitrella_patens/Info/Index, accessed on 12 May 2021), *S. moellendorffii* (http://plants.ensembl.org/Selaginella_moellendorffii/Info/Index, accessed on 18 May 2021), *P. abies* (ftp://plantgenie.org/Data/ConGenIE/Picea_abies/v1.0/, accessed on 20 May 2021), *A. trichopoda* (https://plants.ensembl.org/Amborella_trichopoda/Tools/Blast, accessed on 23 May 2021), *A. thaliana* (http://plants.ensembl.org/Arabidopsis_thaliana/Info/Index, accessed on 2 May 2021), and *O. sativa* (https://plants.ensembl.org/Oryza_sativa/Info/Index, accessed on 4 May 2021). We used a relatively strict criterion to collect ABCs with high-quality sequences. Subsequently, the sequences were searched against the conserved protein domain database [28], SMART [29], and PFAM [30] to validate their reliability. Protein sequences without signature domain CDD (1000606, 1000096, 1000085, 1002808, 11441177, 11477396, 11477521) or PFAM (PF00664, PF12698, PF01061, PF12679, PF19055, PF12730, PF13346, PF12848, PF00005) were discarded.

### 2.2. Phylogenetic Analysis of Gene Families

For each subfamily, multiple alignments of candidate proteins were performed using MAFFT version 7 with the G-INS-i algorithm [31], followed by manual editing in MEGA 7 software [32]. Only positions that were unambiguously aligned were included in the further analyses. Neighbor-joining (NJ) phylogenetic trees were constructed using MEGA 7 software based on the multiple alignment of candidate proteins. To determine the statistical reliability, bootstrap analysis was conducted using 1000 replicates with the p-distance and pairwise deletion. In addition, Maximum Likelihood (ML) phylogenetic trees were constructed using IQ-tree with 1000 replicates to validate the NJ results [33]. 

### 2.3. Gene Expression Analysis

*M. viride* expression datasets, with accession numbers GSE123852 [24], were downloaded from the NCBI Gene Expression Omnibus (GEO). A gene expression heatmap was created using R (https://www.r-project.org/, accessed on 18 March 2021) package “pheatmap”.

## 3. Results and Discussion

### 3.1. Identification of ABC Transporters in Green Plants

**Figure 1:**
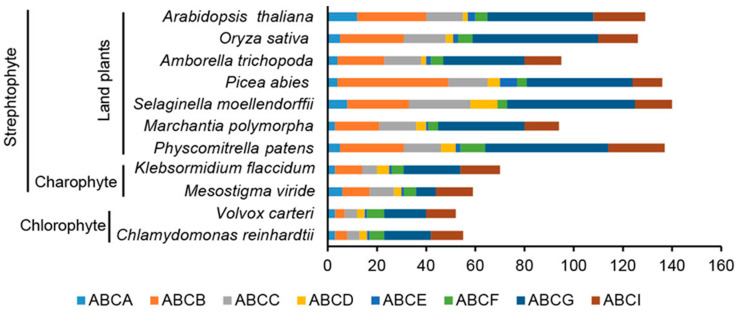
Number of ABC proteins from 11 evolutionarily representative plant species. Each subfamily (ABCA, ABCB, ABCC, ABCD, ABCE, ABCF, ABCG, and ABCI) is labeled with a different color.

To investigate the origin and evolution of the ABC family in plants, we carried out a genome-wide survey of ABC proteins from 11 representative species of chlorophyte (*C.reinhardtii* and *V. carteri*), charophyte (*M. viride* and *K. flaccidum*), bryophyte (*P. patens* and *M. polymorpha*), lycophyte (*S. moellendorffii*), gymnosperm (*P. abies*), basal angiosperm (*A. trichopoda*), monocot (*O. sativa*), and eudicot (*A. thaliana*). After removing incomplete and/or redundant sequences and alternative splice variants, we identified a total of 1093 ABC proteins in the 11 species (Figure 1). Based on the domain annotations, these proteins belong to eight ABC subfamilies: ABCA, ABCB, ABCC, ABCD, ABCE, ABCF, ABCG, and ABCI. Interestingly, we identified 59 ABCs in *M. viride* that were distributed in all subfamilies, 55 of them were supported by RNA-seq data and their expression was dynamic under different environmental condition (Supplementary Figure S1) [24], suggesting most of the ABC genes in *M. viride* were functional. Notably, the eight ABC subfamilies also included chlorophyte green algae, suggesting that ABC subfamily diversification may have evolved and functional in the common ancestor of green plants. Comparing the number of ABC proteins among these species, we found that the number was largely increased from green algae (water) to land plants, coinciding with the functional diversification of ABC transporters in land plants [15,16].

### 3.2. Evolutionary Analyses of ABC Subfamilies

We further performed phylogenetic reconstruction of each subfamily to better understand the evolutionary relationships of ABC transporters and investigate their origin. Considering no standard nomenclature for the classified clades within each subfamily, we “group” to name each clade.

#### 3.2.1. ABCA Subfamily

**Figure 2:**
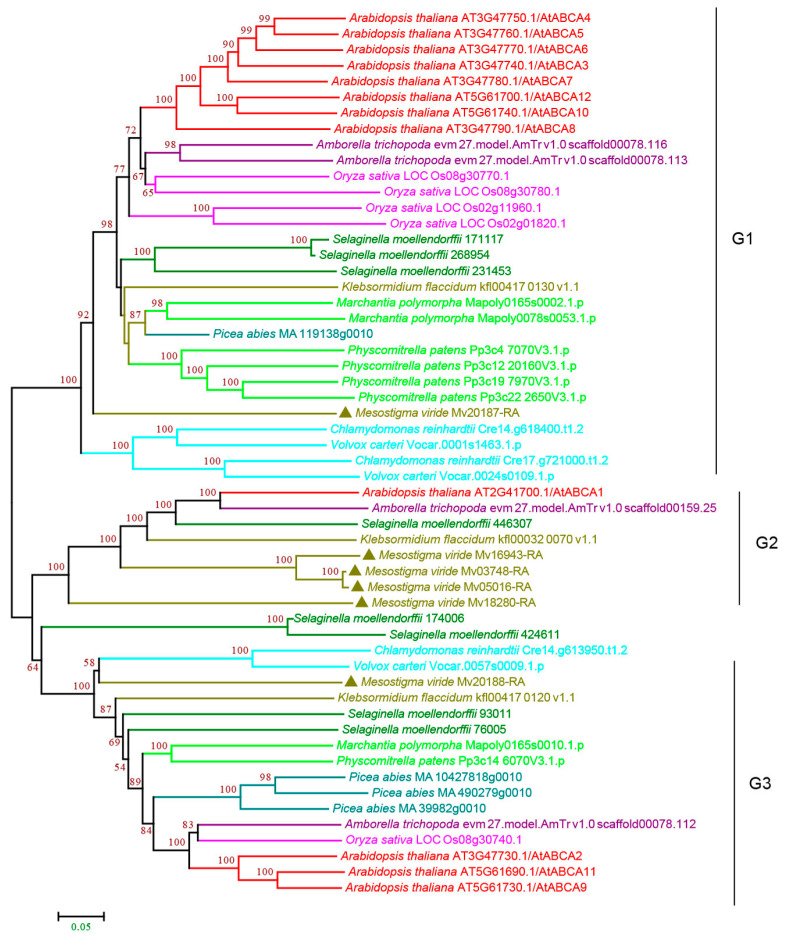
Phylogenetic analysis of ABCA subfamily proteins from 11 evolutionarily representative plant species. A neighbor-joining (NJ) tree was generated using MEGA7 with 1000 bootstrap replicates, and bootstrap values >50% are shown on the branches. The *M. viride* proteins are highlighted by triangles.

Subfamily A consists of forward-oriented (TMD-NBD) transporters and is mainly involved in the manipulation of metabolic and signaling lipids in organisms [34,35]. The plant ABCA subfamily contains one full-size ABCA, named AtABCA1, which is the only full-size ABCA protein and is the largest ABC protein, and several half-size ABCAs that are also called ABC two homologues (ATH) [27,36]. Our analysis showed that the plant ABCA subfamily proteins can be classified into three groups, G1–G3, with strong bootstrap support. The G1 and G3 proteins were half-size ABCAs that were found in all the species examined. G1 proteins were present as multiple copies in most species except for a single copy in *P. abies*, *K. flaccidum,* and *M. viride* (Figure 2 and Supplementary Figure S2), showing a trend of gene expansion during evolution. The G2 proteins contain full-size ABCAs (Figure 2 and Supplementary Figure S2). Interestingly, we observed four copies in the single-cell green algae *M. viride* and one copy in *K. flaccidum*, *S. moellendorffii,* and *A. thaliana,* but not in many species, including rice and early land plants, suggesting multiple losses of G2 proteins during evolution. G3 proteins were present as a single copy in most species except for multiple copies in *P. abies*, *S. moellendorffii,* and *A. thaliana*. It should be noted that G1–G3 proteins were all found in *M. viride* (Figure 2 and Supplementary Figure S2), suggesting that all three groups have an ancient origin. 

#### 3.2.2. ABCB Subfamily

**Figure 3:**
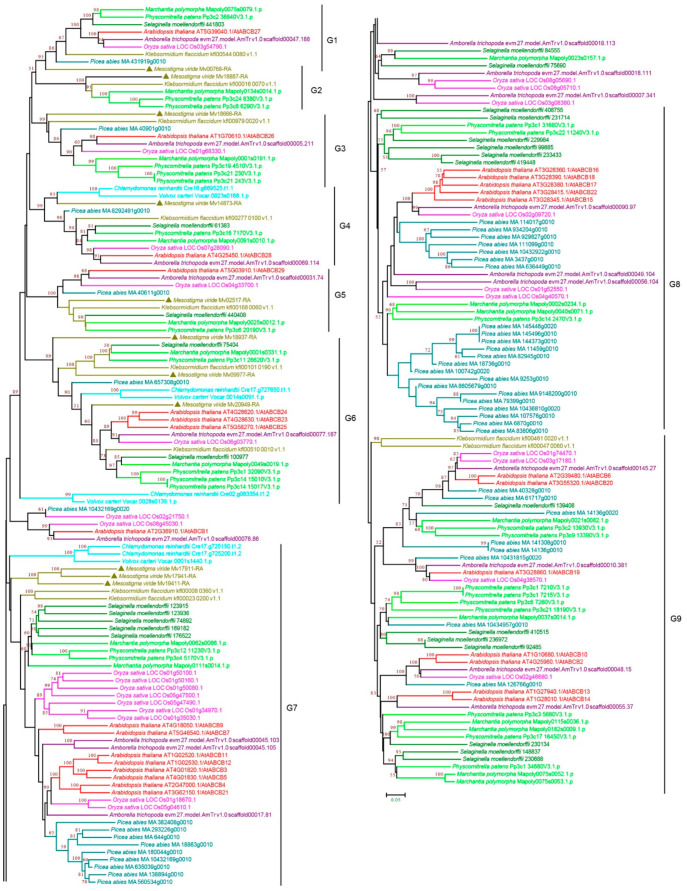
Phylogenetic analysis of ABCB subfamily proteins from 11 evolutionarily representative plant species. A neighbor-joining (NJ) tree was generated using MEGA7 with 1000 bootstrap replicates, and bootstrap values >50% are shown on the branches. The *M. viride* proteins are highlighted by triangles.

ABCB proteins are functionally diverse and represent the second largest group of ABC proteins in plants. This subfamily includes full-size proteins, multidrug resistance protein (MDR) or P-glycoprotein (PGP) [37,38], and half-size proteins transporter associated with antigen processing (TAP), ABC transporter of mitochondria (ATM), and lipid A-like exporter putative (LLP) [27,39,40,41]. Our phylogenetic analysis revealed nine groups, G1-G9, and a few orphans (Figure 3 and Supplementary Figure S3). The high bootstrap value (89 NJ/100 ML) suggests that G1–G6 are closely related, and the seven groups (G1-G7) are likely more ancient because proteins were found in *C. reinhardtii*, *V. carteri,* and *M. viride*. Notably, G2 proteins were only present in charophyte green algae and early diverging land plants (Figure 3 and Supplementary Figure S3), suggesting that they were lost in vascular plants. G7, G8, and G9 contain more ABCBs, and many duplications were observed, especially the duplication within *P. abies* in G7 and G8, which contributed to the relatively bigger subgroup size. Interestingly, G7-G9 contained only three chlorophyte proteins (Figure 3 and Supplementary Figure S3), and many ABCBs, such as ABCB19, ABCB4, ABCB21, ABCB11, ABCB14, and ABCB15, which are involved in auxin transport and plant development, were found in G7–G9, consistent with auxin transport evolution in Streptophyta [42,43,44,45,46,47,48].

#### 3.2.3. ABCC Subfamily

**Figure 4:**
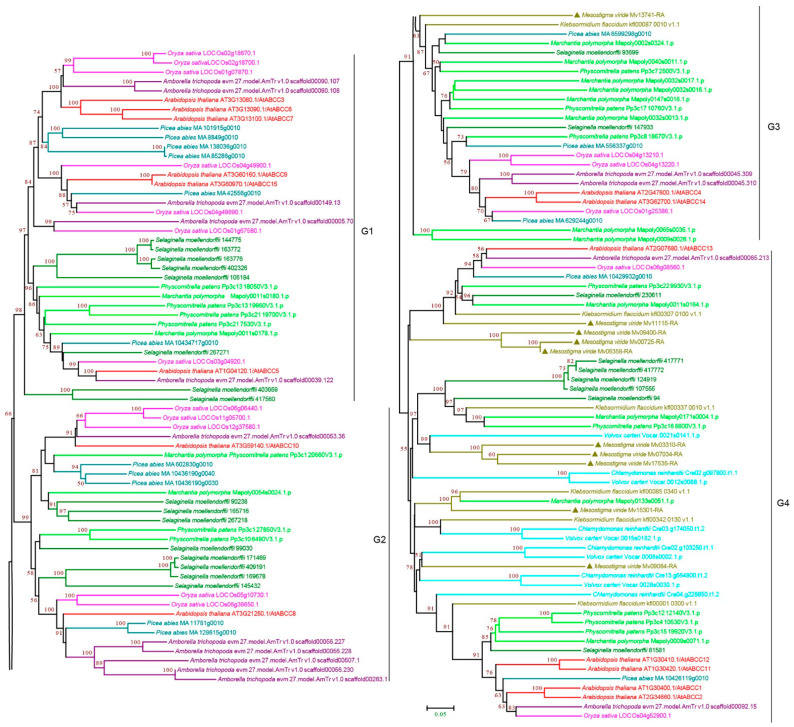
Phylogenetic analysis of ABCC subfamily proteins from 11 evolutionarily representative plant species. A neighbor-joining (NJ) tree was generated using MEGA7 with 1000 bootstrap replicates, and bootstrap values >50% are shown on the branches. The *M. viride* proteins are highlighted by triangles.

ABCC subfamily proteins, also known as multidrug resistance-associated proteins (MRPs), are full-length transporters in plants, and most of them contain an additional N-terminal hydrophobic region [11,49]. In plants, ABCCs are localized to the vacuolar membrane and plasma membrane [18,50]. They transport not only plant-derived compounds but also chlorophyll degradation metabolites and phytochelatins [50,51]. Therefore, ABCCs have thus far been described as transporters involved in internal detoxification [15]. Our phylogenetic analysis classified ABCCs into four subgroups: G1–G4 (Figure 4 and Supplementary Figure S4). The presence of ABCCs in green algae allowed us to identify the ancestral versions. We found green algae ABCCs in G3 and G4, and the chlorophytes *C. reinhardtii* and *V. carteri* were only present in G4. Together with all the protein sequences of G1 and G2 were from land plants (Figure 4 and Supplementary Figure S4), suggesting that these two groups, particularly G4, are more ancient, and G1 and G2 may have evolved after plants colonized land or green algae lost G1 and G2 proteins during evolution. Interestingly, within the subgroups, G4 contained nine copies of ABCCs in *M. viride*, which was the highest copy number among all species, suggesting that duplication of ABCC occurred at the base of Charophyta. Consistently, we observed multiple copies of ABCC in early land plants (Figure 4 and Supplementary Figure S4). However, the copy number was not significantly increased in seed plants, which have generally undergone whole genome duplication (WGD), suggesting a possible purifying selection in ABCCs during evolution.

#### 3.2.4. ABCD Subfamily

**Figure 5:**
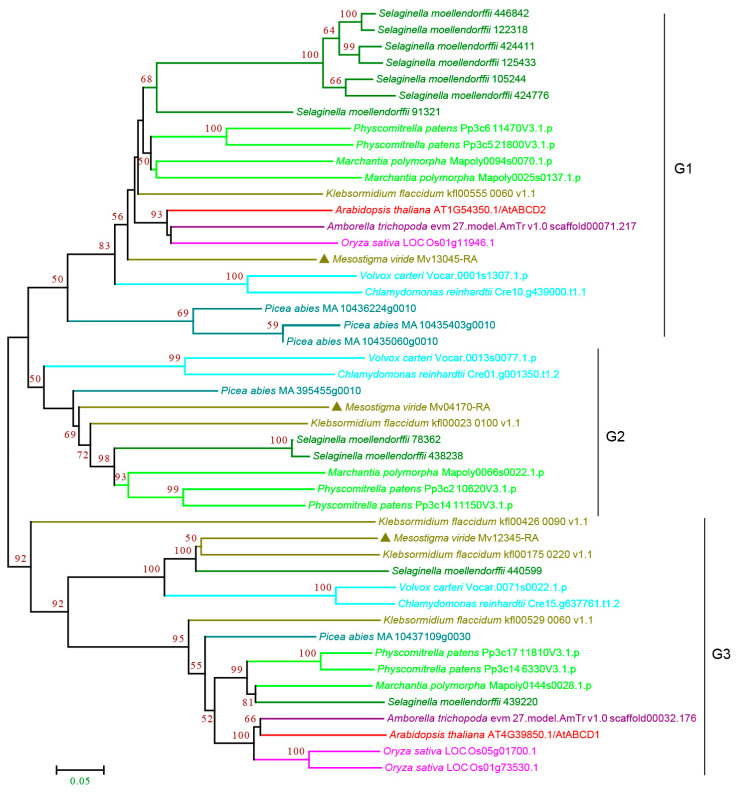
Phylogenetic analysis of ABCD subfamily proteins from 11 evolutionarily representative plant species. A neighbor-joining (NJ) tree was generated using MEGA7 with 1000 bootstrap replicates, and bootstrap values >50% are shown on the branches. The *M. viride* proteins are highlighted by triangles.

The ABCD family contains predominantly half-size proteins with the orientation TMD-NBD, also known as PMPs (peroxisomal membrane proteins). They are associated with peroxisomal import of fatty acids [52,53,54,55]. The phylogenetic analysis identified three groups: G1–G3 (Figure 5 and Supplementary Figure S5). *M. viride* and chlorophyte green algae protein were observed in all groups, indicating the ancient origin of ABCDs. We found that every species examined contains half-size G1 and full-size G3 proteins. Interestingly, the copy number increased during the transition from water to land and subsequently decreased during the transition to flowering plants (Figure 5 and Supplementary Figure S5). However, we found G2 proteins in most species except *A. thaliana*, *O. sativa,* and *P. abies*, suggesting that G2 was lost in flowering plants from the basal angiosperm.

#### 3.2.5. ABCE and ABCF Subfamilies

**Figure 6:**
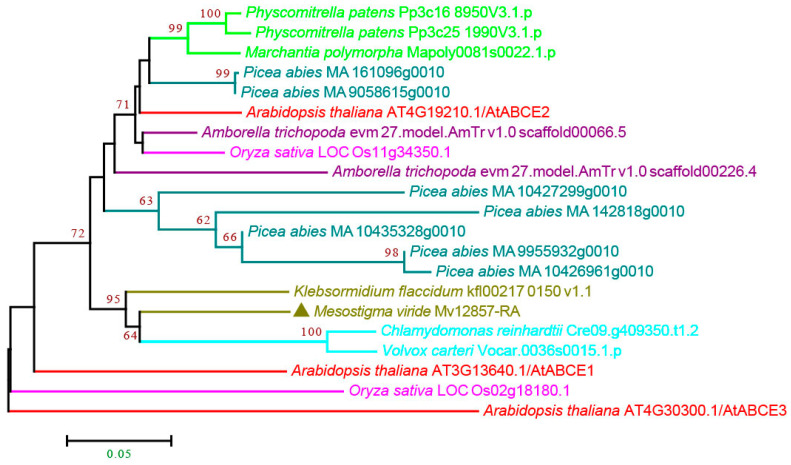
Phylogenetic analysis of ABCE subfamily proteins from 11 evolutionarily representative plant species. A neighbor-joining (NJ) tree was generated using MEGA7 with 1000 bootstrap replicates, and bootstrap values >50% are shown on the branches. The *M. viride* proteins are highlighted by triangles.

Atypical ABC transporters are present in the ABCE and ABCF subfamilies, which lack TMDs and consist of two NBDs [4,13]. ABCE was first identified as an RNase L inhibitor (RLI) in Homo sapiens and is a highly evolutionarily conserved protein [56]. In plants, the ABCE was the smallest subfamily among all the ABC subfamilies (Figure 1), and ABCEs were found in all the species, including *M. viride* (Figure 6 and Supplementary Figure S5). We found that most species contain 1–3 copies, except *P. abies*, which has seven copies of ABCEs, suggesting that the function of ABCEs is highly conserved and possibly redundant in very few species.

**Figure 7:**
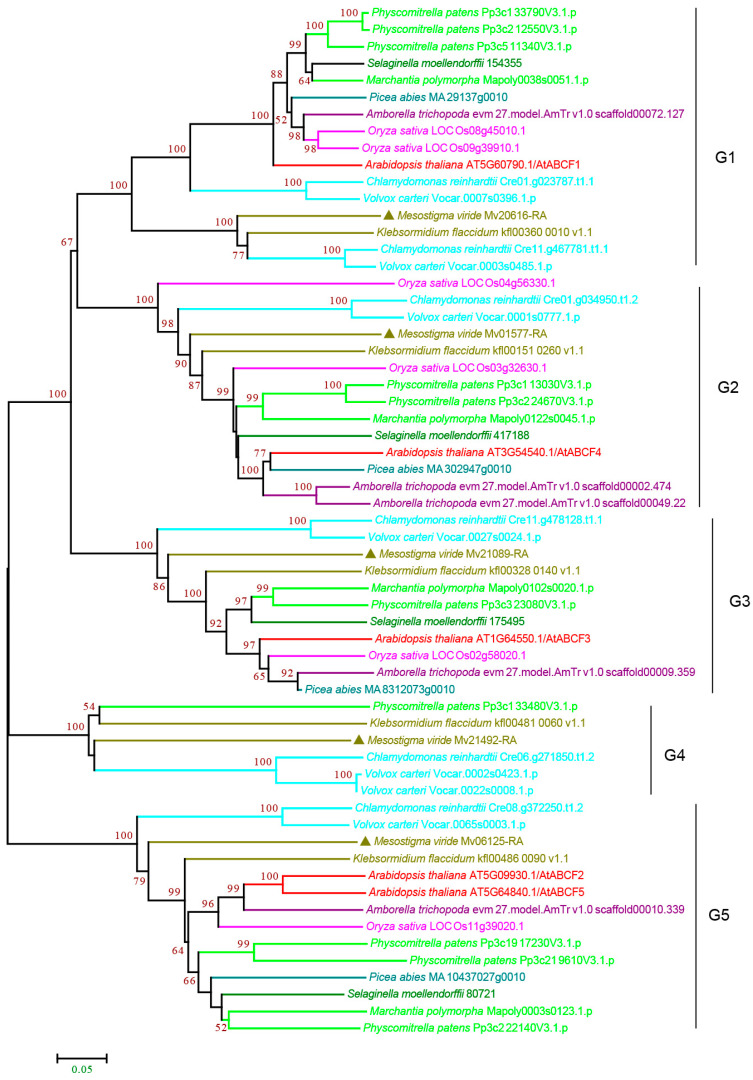
Phylogenetic analysis of ABCF subfamily proteins from 11 evolutionarily representative plant species. A neighbor-joining (NJ) tree was generated using MEGA7 with 1000 bootstrap replicates, and bootstrap values >50% are shown on the branches. The *M. viride* proteins are highlighted by triangles.

In humans and yeast, ABCF proteins are involved in ribosome assembly and protein translation [57]. Our phylogenetic analysis classified the plant ABCFs into five groups: G1–G5 (Figure 7 and Supplementary Figure S7). The presence of green algae ABCFs suggested that all the groups have an ancient origin. We found that all the species contain G1–G3 and G5 proteins. However, G4 only included green algae and *P. patens* (Figure 7 and Supplementary Figure S7), suggesting that G4 was lost in most land plants. Notably, for each group, we did not find a large copy number of ABCFs, and all the species contained 1–3 copies (Figure 7 and Supplementary Figure S7).

#### 3.2.6. ABCG Subfamily

**Figure 8:**
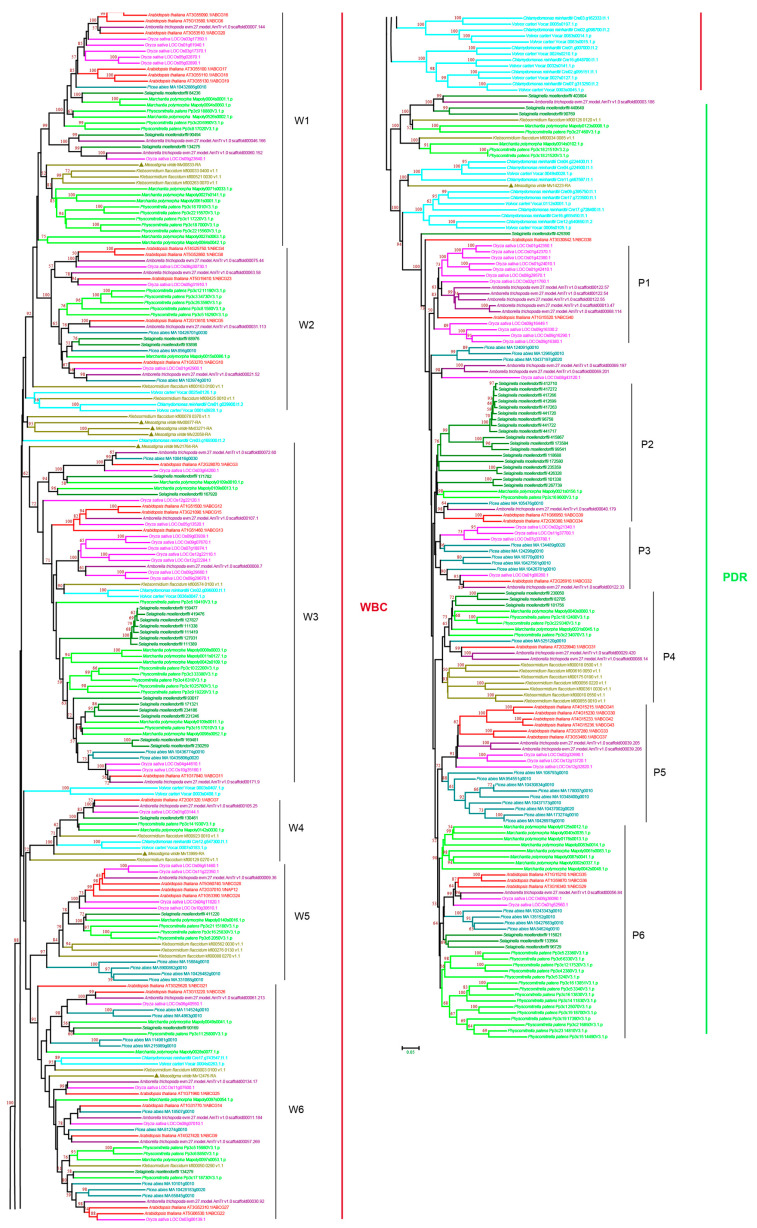
Phylogenetic analysis of ABCG subfamily proteins from 11 evolutionarily representative plant species. A neighbor-joining (NJ) tree was generated using MEGA7 with 1000 bootstrap replicates, and bootstrap values >50% are shown on the branches. ABCGs include two major groups: the white–brown complex (WBC), and pleiotropic drug resistance proteins (PDRs). The *M. viride* proteins are highlighted by triangles.

ABCG is the largest ABC transporter subfamily in plants and has an NBD-TMD reverse domain architecture [6,7]. ABCGs have been reported to be involved in the transport of various secondary metabolites in plants [6], such as detoxification materials, hormones, and lipids [16,58,59,60,61]. The plant ABCGs include two major groups: the white–brown complex (WBC), named after *Drosophila melanogaster*, which comprises half-size ABCG proteins, and pleiotropic drug resistance proteins (PDRs), named after the yeast prototype, which contain a large group of full-sized ABCG proteins [62,63]. Our phylogenetic analysis revealed six WBC groups, W1–W6, and one PDR group (Figure 8 and Supplementary Figure S8). For the WBC groups, *M. viride* was observed in W1–W3 and W5–W6, and the copy number of ABCG was largely expanded in multicellular plants. For W3, we observed 11 ABCGs in *S. moellendorffii*, representing the largest number within the WBC groups (Figure 8). The group of PDRs was highly supported by the bootstrap value, suggesting that PDRs have evolved from a common ancestor with full size. A single copy of PDR was present in *M. viride*. It should be noted that in other species, PDRs were largely expanded, and even more expanded than WBCs. The copy number of PDRs ranged from nine for *K. flaccidum* to 22 for *S. moellendorffii* (Figure 8 and Supplementary Figure S8). Based on the topology and bootstrap support, the PDR contains six smaller clusters, P1–P6, and the copy number is variable between species. For example, most of the rice PDRs were found in P1, and the majority of *P. patens* PDRs were found in P6 (Figure 8 and Supplementary Figure S8), suggesting that duplication may occur within specific species.

#### 3.2.7. ABCI Subfamily

**Figure 9:**
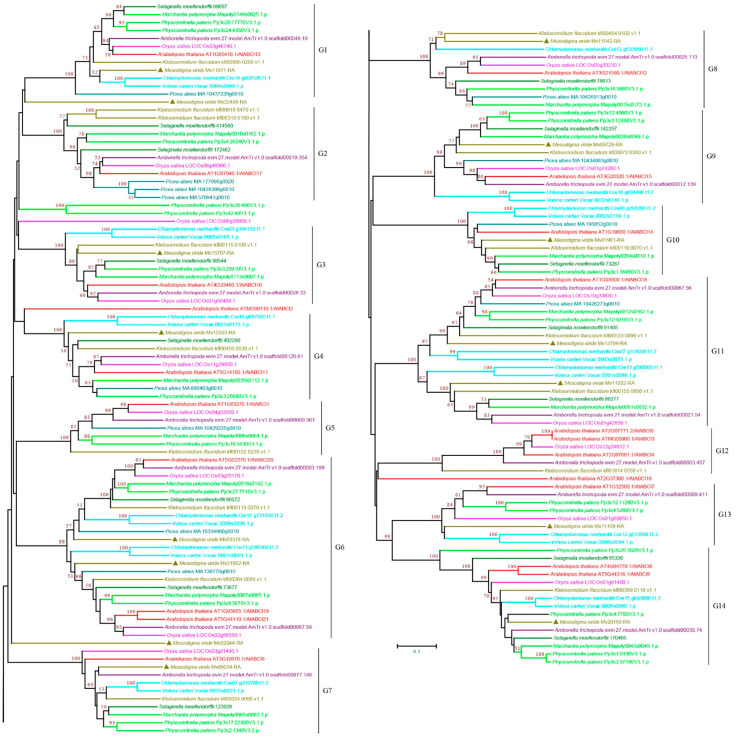
Phylogenetic analysis of ABCI subfamily proteins from 11 evolutionarily representative plant species. A neighbor-joining (NJ) tree was generated using MEGA7 with 1000 bootstrap replicates, and bootstrap values >50% are shown on the branches. The *M. viride* proteins are highlighted by triangles.

The ABCH subfamily is found in animals but not in plants. Instead, plants contain a group of nonintrinsic ABC proteins, named ABCIs, which have only NBD domains [11,18,64,65]. The phylogenetic analysis classified the ABCIs into 14 groups, G1–G14, with high bootstrap support (Figure 9 and Supplementary Figure S9). *M. viride* ABCIs were found in 11 of the 14 groups and were only absent in G2, G5, and G12, suggesting that most groups have a deep origin and that the expansion of ABCI may have occurred in the common ancestor. In contrast to the high number of copies and frequent duplications of other subfamilies, one or two copies of ABCIs were usually found in the species examined for each group, and the highest copy number was three, which was found in *Arabidopsis* of G6 and G12, and in *P. abies* of G2. Notably, ABCI was not identified in many species in G5 and G12, which may indicate multiple gene loss events during evolution.

## 4. Conclusions

As key players in plant growth and development, ABC transporters are of interest for their potential applications in agriculture. Here, we report the identification and evolution of ABCs in *Mesostigma viride*. Our comprehensive and updated phylogenetic analysis provides new insights into understanding the evolutionary mechanisms underlying the origin and expansion of plant ABC transporters and provides a valuable resource for investigating the physiological functions of ABC genes.

## Data Availability

Data available on request from the author.

## Supplementary Materials

The following supporting information can be downloaded at: https://www.mdpi.com/article/10.3390/cimb44040112/s1, Figure S1: Expression profiles of ABC genes in *M. viride*. The genes IDs are on the right. The different environmental conditions used for expression analysis are indicated at the bottom of each column. Grey color indicates no expression. Figure S2: Phylogenetic analysis of ABCA subfamily proteins from 11 evolutionarily representative plant species. A Maximum Likelihood (ML) tree was generated using IQ-tree with 1000 bootstrap replicates, and bootstrap values >50% are shown on the branches. The *M. viride* proteins are highlighted by triangles. Figure S3: Phylogenetic analysis of ABCB subfamily proteins from 11 evolutionarily representative plant species. A Maximum Likelihood (ML) tree was generated using IQ-tree with 1000 bootstrap replicates, and bootstrap values >50% are shown on the branches. The *M. viride* proteins are highlighted by triangles. Figure S4: Phylogenetic analysis of ABCC subfamily proteins from 11 evolutionarily representative plant species. A Maximum Likelihood (ML) tree was generated using IQ-tree with 1000 bootstrap replicates, and bootstrap values >50% are shown on the branches. The *M. viride* proteins are highlighted by triangles. Figure S5: Phylogenetic analysis of ABCD subfamily proteins from 11 evolutionarily representative plant species. A Maximum Likelihood (ML) tree was generated using IQ-tree with 1000 bootstrap replicates, and bootstrap values >50% are shown on the branches. The *M. viride* proteins are highlighted by triangles. Figure S6: Phylogenetic analysis of ABCE subfamily proteins from 11 evolutionarily representative plant species. A Maximum Likelihood (ML) tree was generated using IQ-tree with 1000 bootstrap replicates, and bootstrap values >50% are shown on the branches. The *M. viride* proteins are highlighted by triangles. Figure S7: Phylogenetic analysis of ABCF subfamily proteins from 11 evolutionarily representative plant species. A Maximum Likelihood (ML) tree was generated using IQ-tree with 1000 bootstrap replicates, and bootstrap values >50% are shown on the branches. The *M. viride* proteins are highlighted by triangles. Figure S8: Phylogenetic analysis of ABCG subfamily proteins from 11 evolutionarily representative plant species. A Maximum Likelihood (ML) tree was generated using IQ-tree with 1000 bootstrap replicates, and bootstrap values >50% are shown on the branches. ABCGs include two major groups: the white–brown complex (WBC), and pleiotropic drug resistance proteins (PDRs). The *M. viride* proteins are highlighted by triangles. Figure S9: Phylogenetic analysis of ABCI subfamily proteins from 11 evolutionarily representative plant species. A Maximum Likelihood (ML) tree was generated using IQ-tree with 1000 bootstrap replicates, and bootstrap values >50% are shown on the branches. The *M. viride* proteins are highlighted by triangles.
