# Supplementary material for: New Insights into Evolution of the ABC Transporter Family in Mesostigma viride, a Unicellular Charophyte Algae
Source: Curr Issues Mol Biol. 2022 Apr 11;44(4):1646–60. doi: 10.3390/cimb44040112 (PMC9164057; doi:10.3390/cimb44040112)
Supplement: Supplementary file 1 [file cimb-44-00112-s001.zip › Supplementary figure legends.pdf]

Supplementary Figure S1. Expression profiles of ABC genes in *M. viride*. The genes IDs are on the right. The different environmental conditions used for expression analysis are indicated at the bottom of each column. Grey color indicates no expression.

Supplementary Figure S2. Phylogenetic analysis of ABCA subfamily proteins from 11 evolutionarily representative plant species. A Maximum Likelihood (ML) tree was generated using IQ-tree with 1000 bootstrap replicates, and bootstrap values >50% are shown on the branches. The *M. viride* proteins are highlighted by triangles.

Supplementary Figure S3. Phylogenetic analysis of ABCB subfamily proteins from 11 evolutionarily representative plant species. A Maximum Likelihood (ML) tree was generated using IQ-tree with 1000 bootstrap replicates, and bootstrap values >50% are shown on the branches. The *M. viride* proteins are highlighted by triangles.

Supplementary Figure S4. Phylogenetic analysis of ABCC subfamily proteins from 11 evolutionarily representative plant species. A Maximum Likelihood (ML) tree was generated using IQ-tree with 1000 bootstrap replicates, and bootstrap values >50% are shown on the branches. The *M. viride* proteins are highlighted by triangles.

Supplementary Figure S5. Phylogenetic analysis of ABCD subfamily proteins from 11 evolutionarily representative plant species. A Maximum Likelihood (ML) tree was generated using IQ-tree with 1000 bootstrap replicates, and bootstrap values >50% are shown on the branches. The *M. viride* proteins are highlighted by triangles.

Supplementary Figure S6. Phylogenetic analysis of ABCE subfamily proteins from 11 evolutionarily representative plant species. A Maximum Likelihood (ML) tree was generated using IQ-tree with 1000 bootstrap replicates, and bootstrap values >50% are shown on the branches. The *M. viride* proteins are highlighted by triangles.

Supplementary Figure S7. Phylogenetic analysis of ABCF subfamily proteins from 11 evolutionarily representative plant species. A Maximum Likelihood (ML) tree was generated using IQ-tree with 1000 bootstrap replicates, and bootstrap values >50% are shown on the branches. The *M. viride* proteins are highlighted by triangles.

Supplementary Figure S8. Phylogenetic analysis of ABCG subfamily proteins from 11 evolutionarily representative plant species. A Maximum Likelihood (ML) tree was generated using IQ-tree with 1000 bootstrap replicates, and bootstrap values >50% are shown on the branches. ABCGs include two major groups: the white–brown complex (WBC), and pleiotropic drug resistance proteins (PDRs). The *M. viride* proteins are highlighted by triangles.

Supplementary Figure S9. Phylogenetic analysis of ABCI subfamily proteins from 11 evolutionarily representative plant species. A Maximum Likelihood (ML) tree was generated using IQ-tree with 1000 bootstrap replicates, and bootstrap values >50% are shown on the branches. The *M. viride* proteins are highlighted by triangles.
