# Supplementary figures and images for: New Insights into Evolution of the ABC Transporter Family in Mesostigma viride, a Unicellular Charophyte Algae
Source: Curr Issues Mol Biol. 2022 Apr 11;44(4):1646–60. doi: 10.3390/cimb44040112 (PMC9164057; doi:10.3390/cimb44040112)

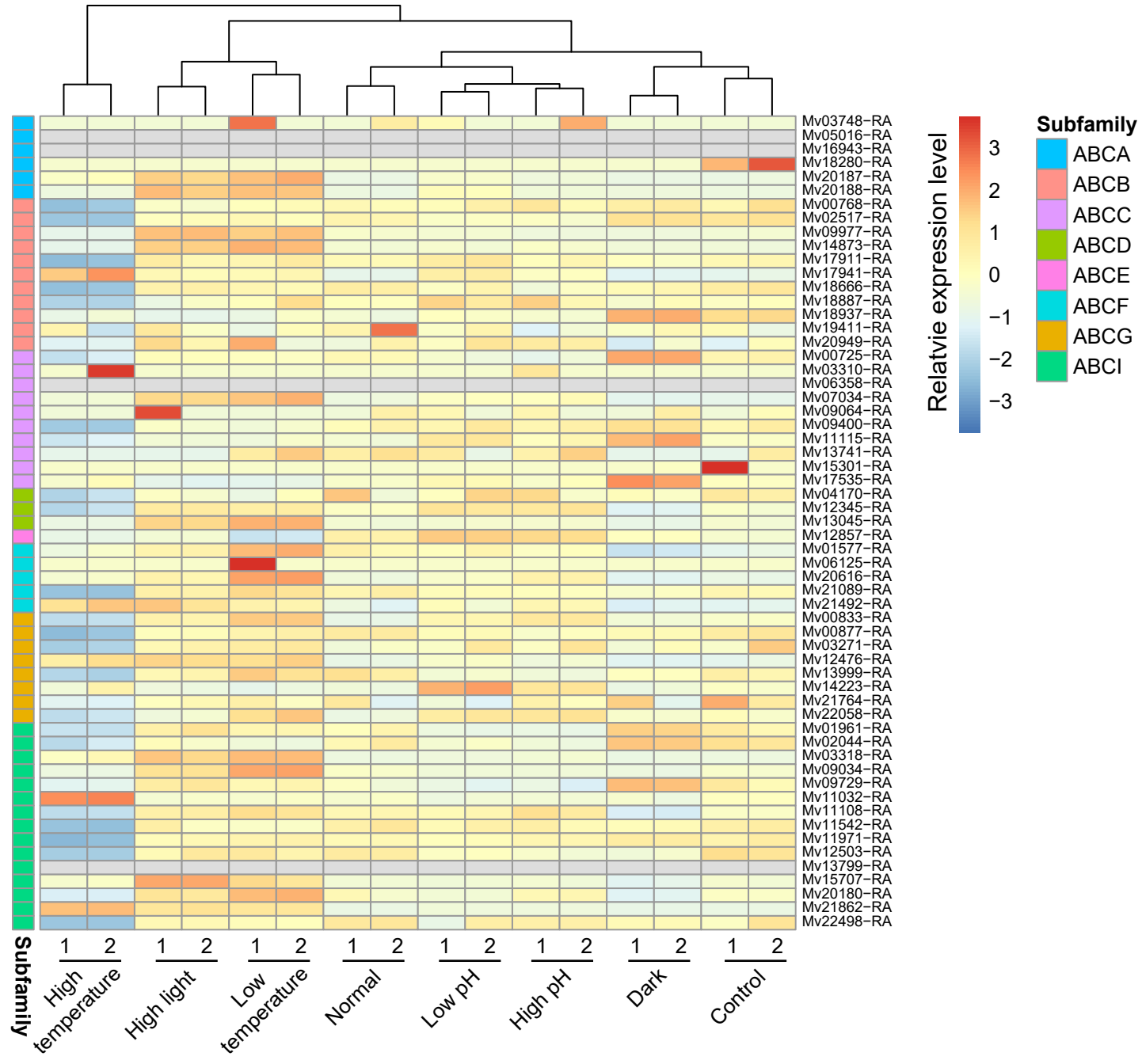

Supplement: Supplementary file 1 [file cimb-44-00112-s001.zip › Figure S1.pdf]

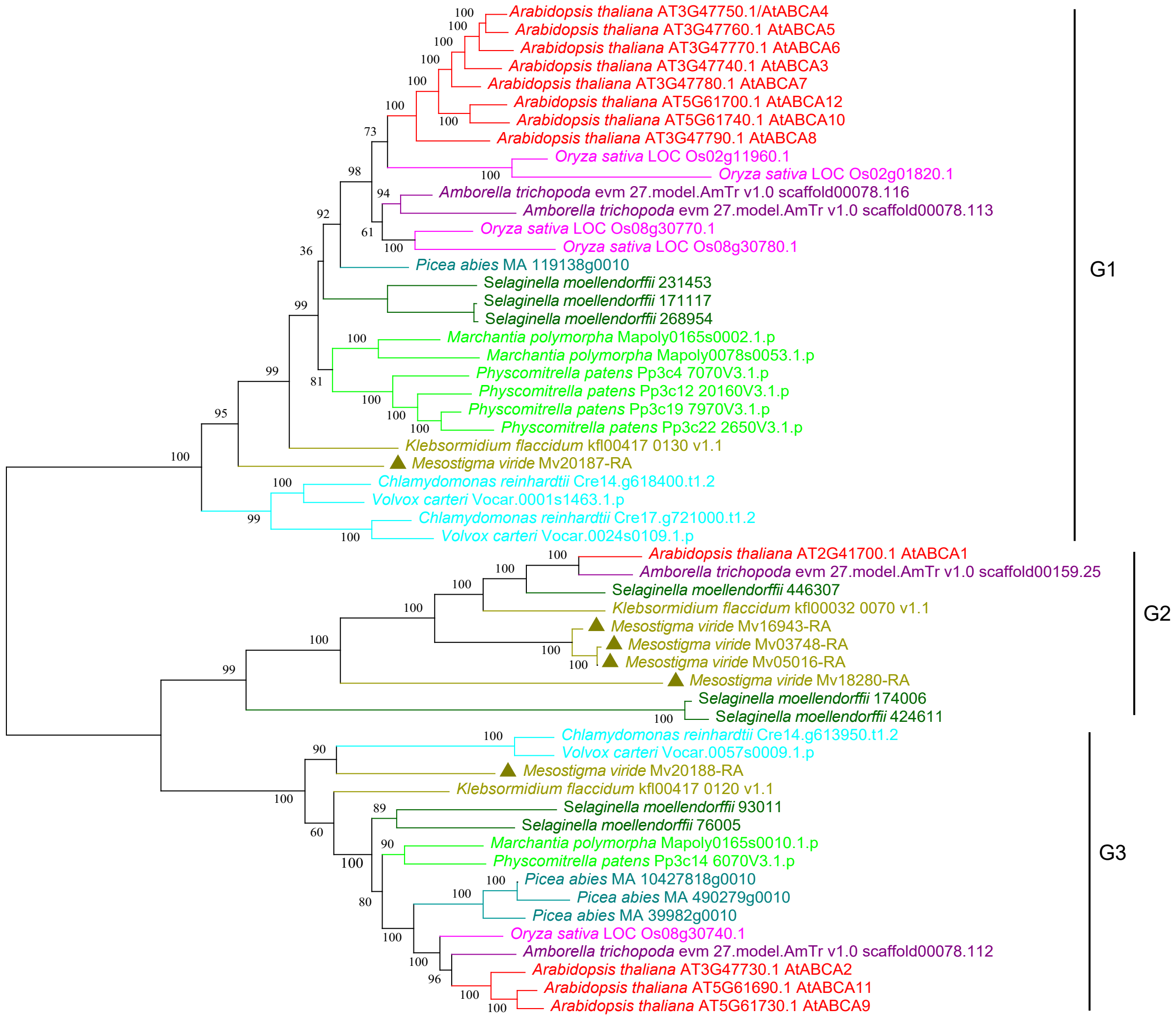

Supplement: Supplementary file 1 [file cimb-44-00112-s001.zip › Figure S2.pdf]

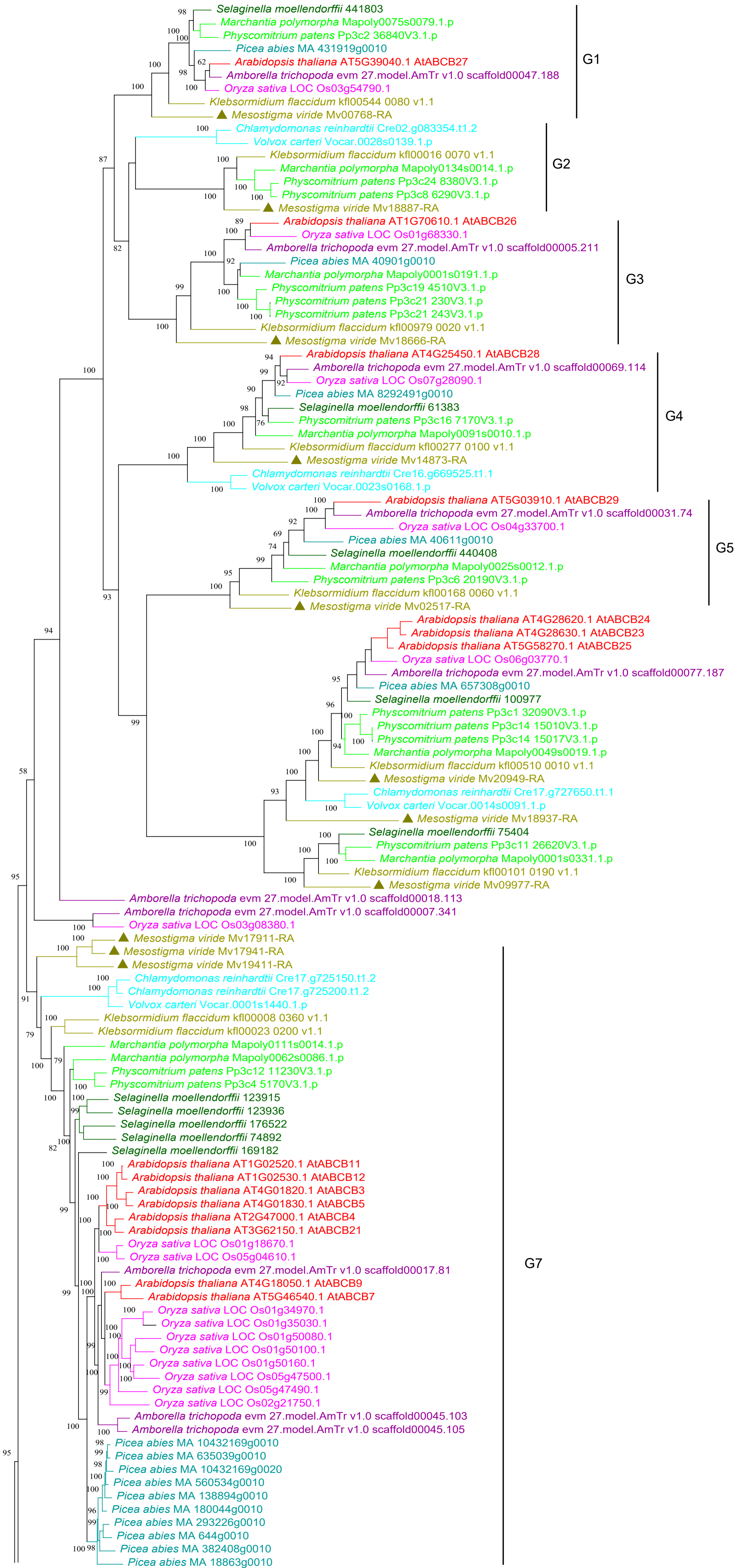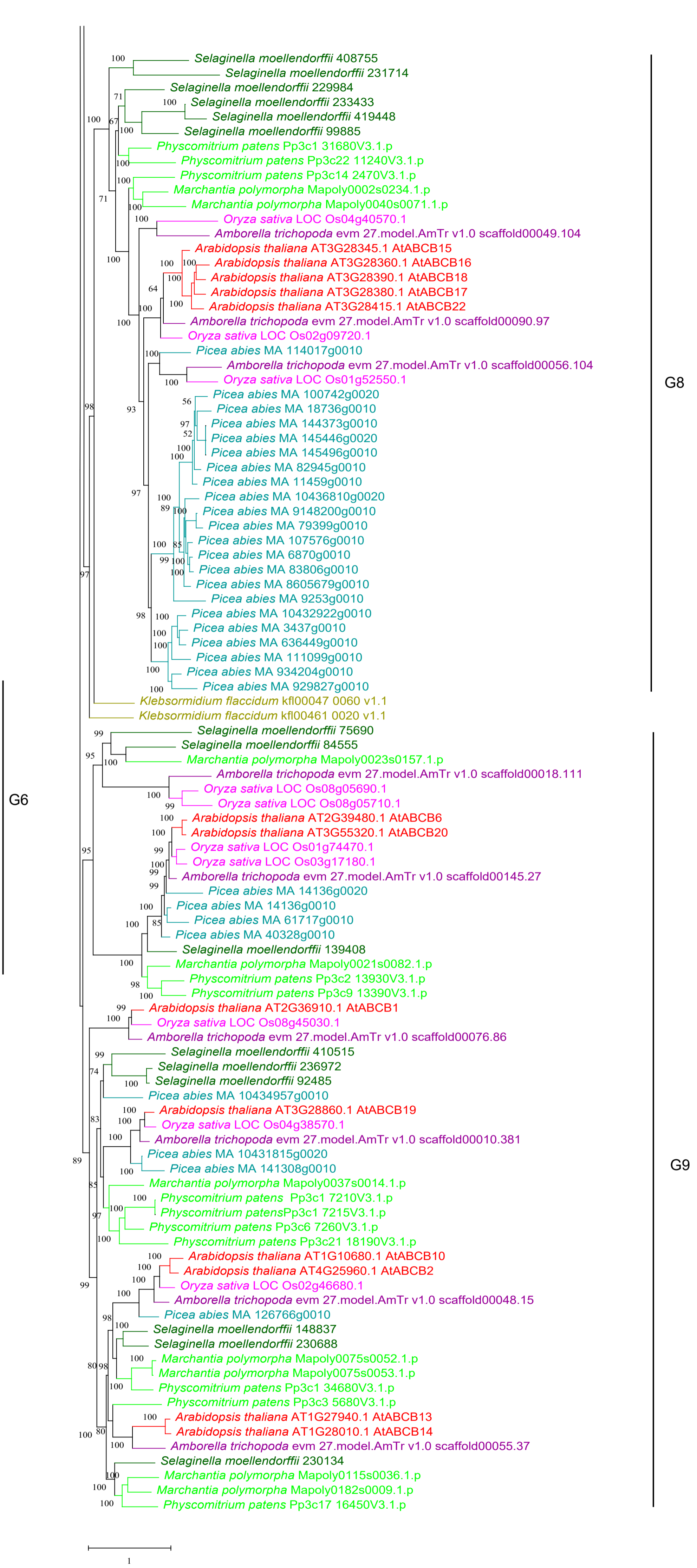

Supplement: Supplementary file 1 [file cimb-44-00112-s001.zip › Figure S3.pdf]

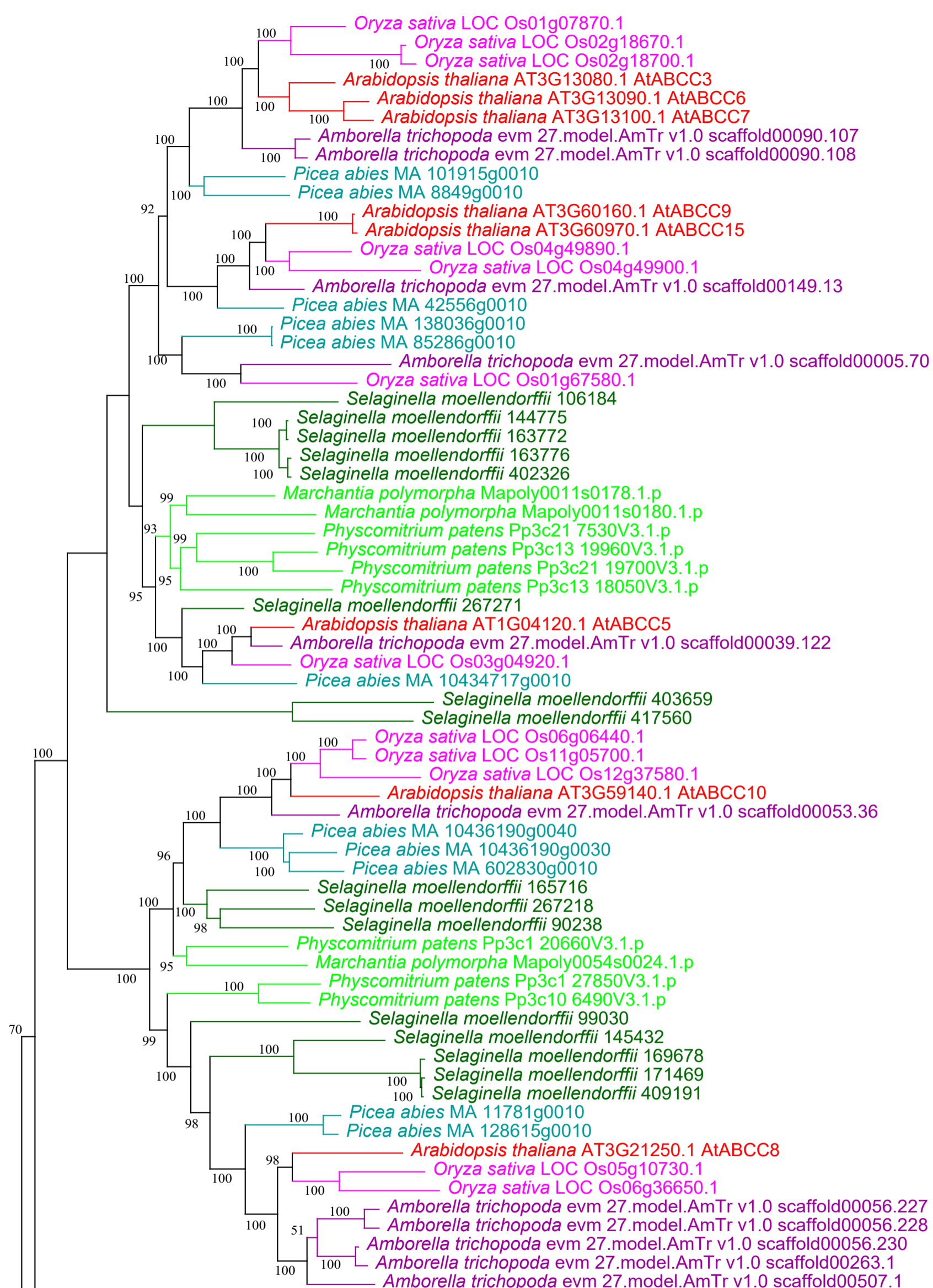

G1

G2

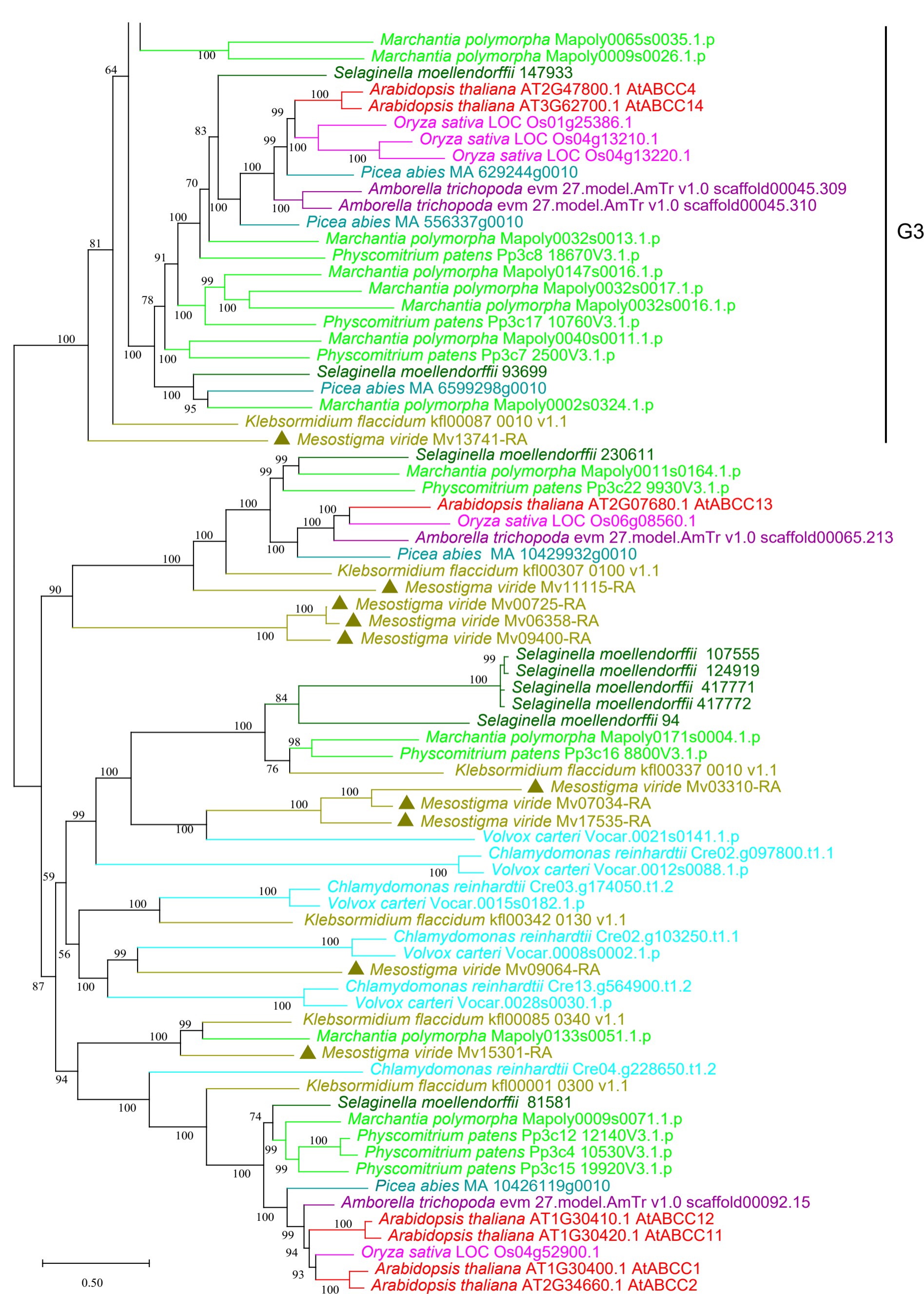

G3

G4

Supplement: Supplementary file 1 [file cimb-44-00112-s001.zip › Figure S4.pdf]

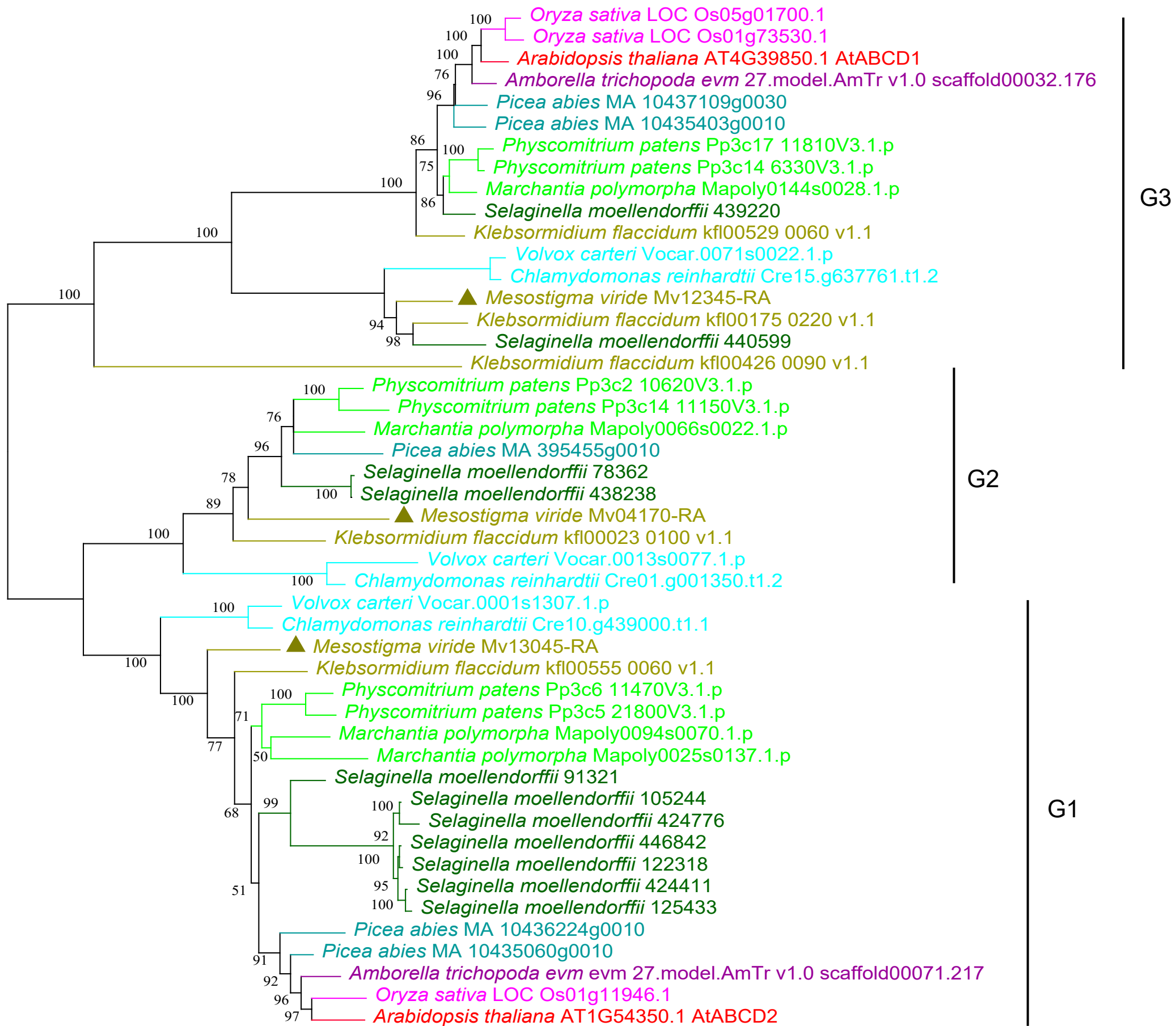

Supplement: Supplementary file 1 [file cimb-44-00112-s001.zip › Figure S5.pdf]

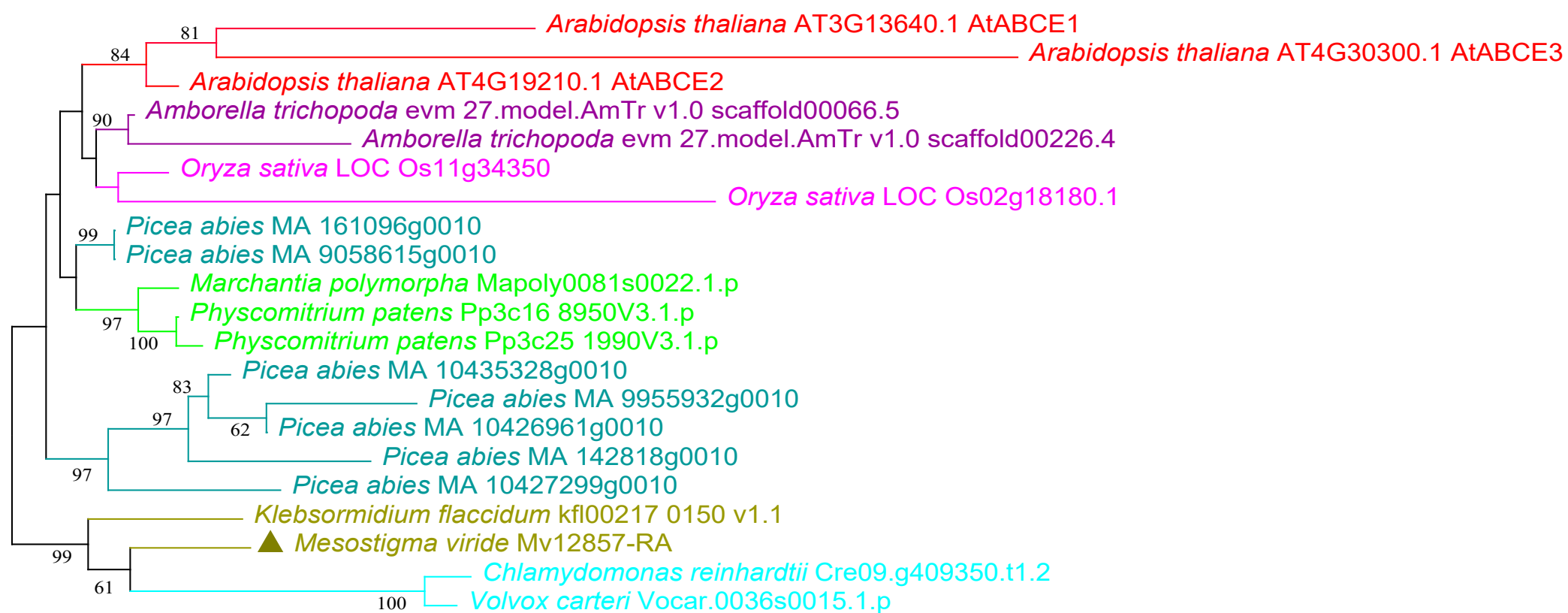

0.10

Supplement: Supplementary file 1 [file cimb-44-00112-s001.zip › Figure S6.pdf]

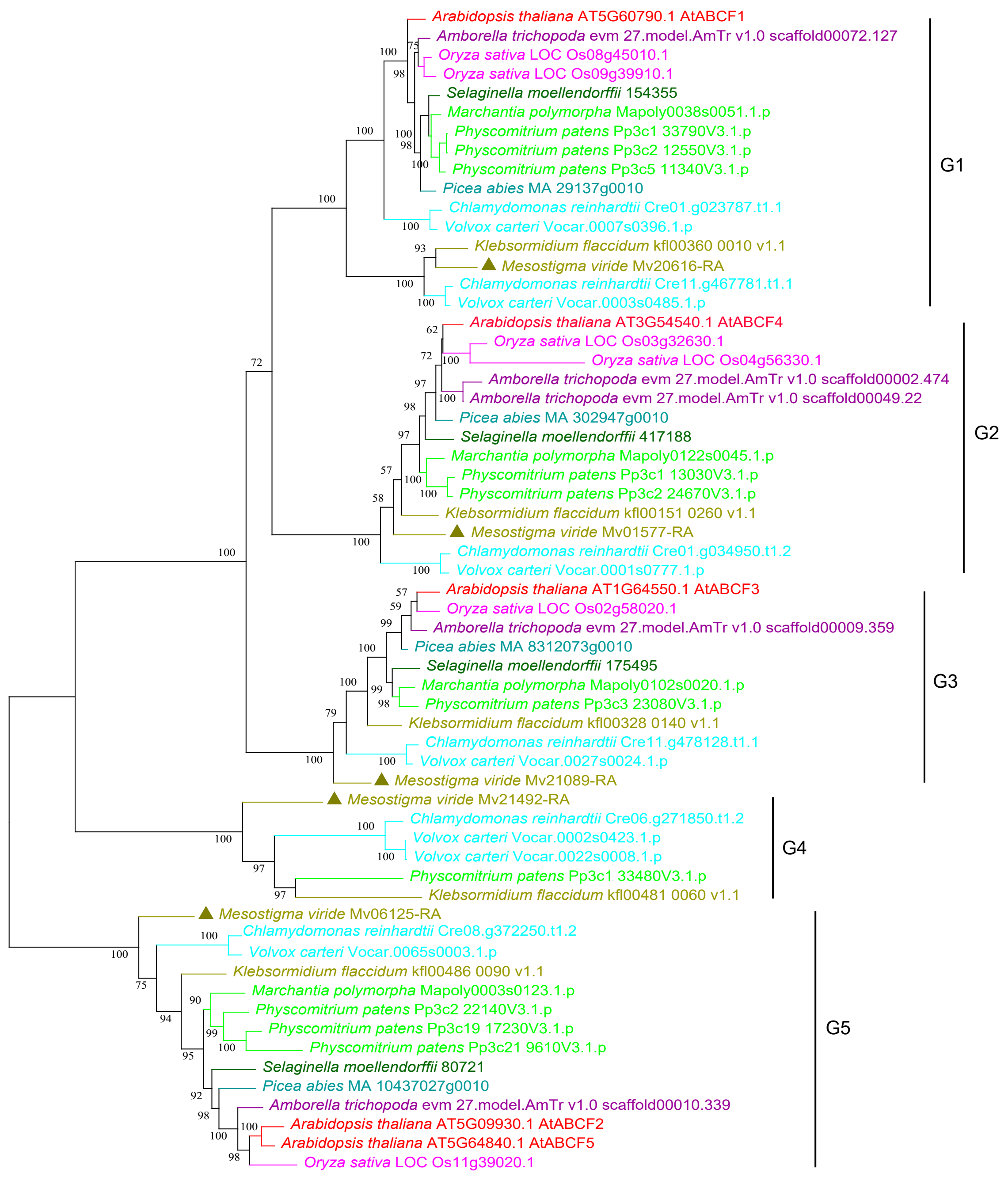

1

Supplement: Supplementary file 1 [file cimb-44-00112-s001.zip › Figure S7.pdf]

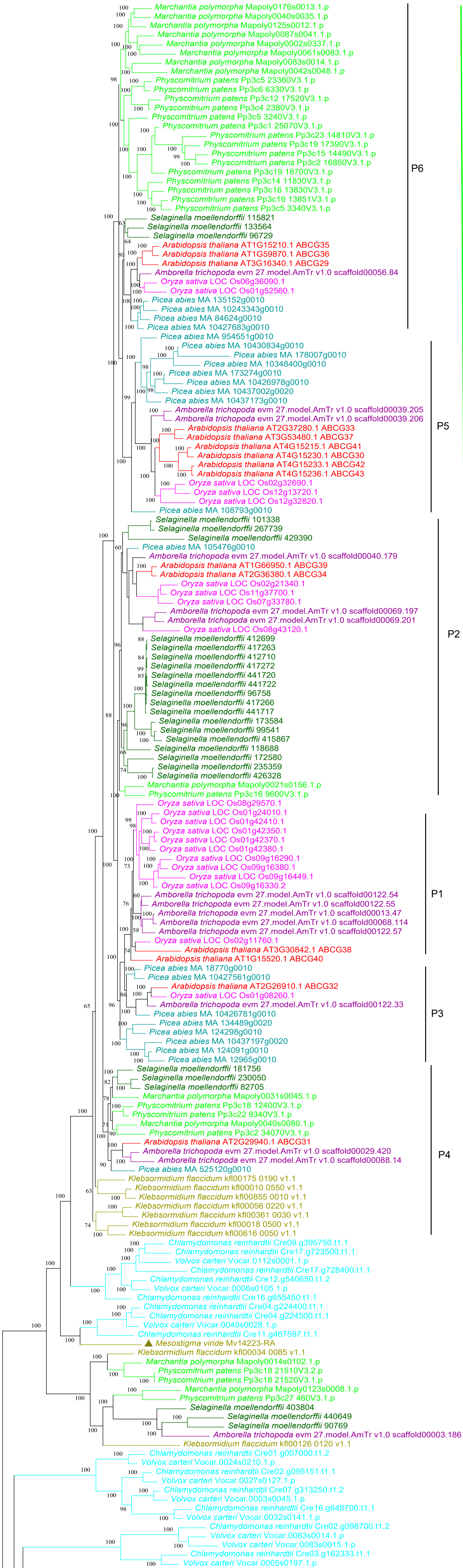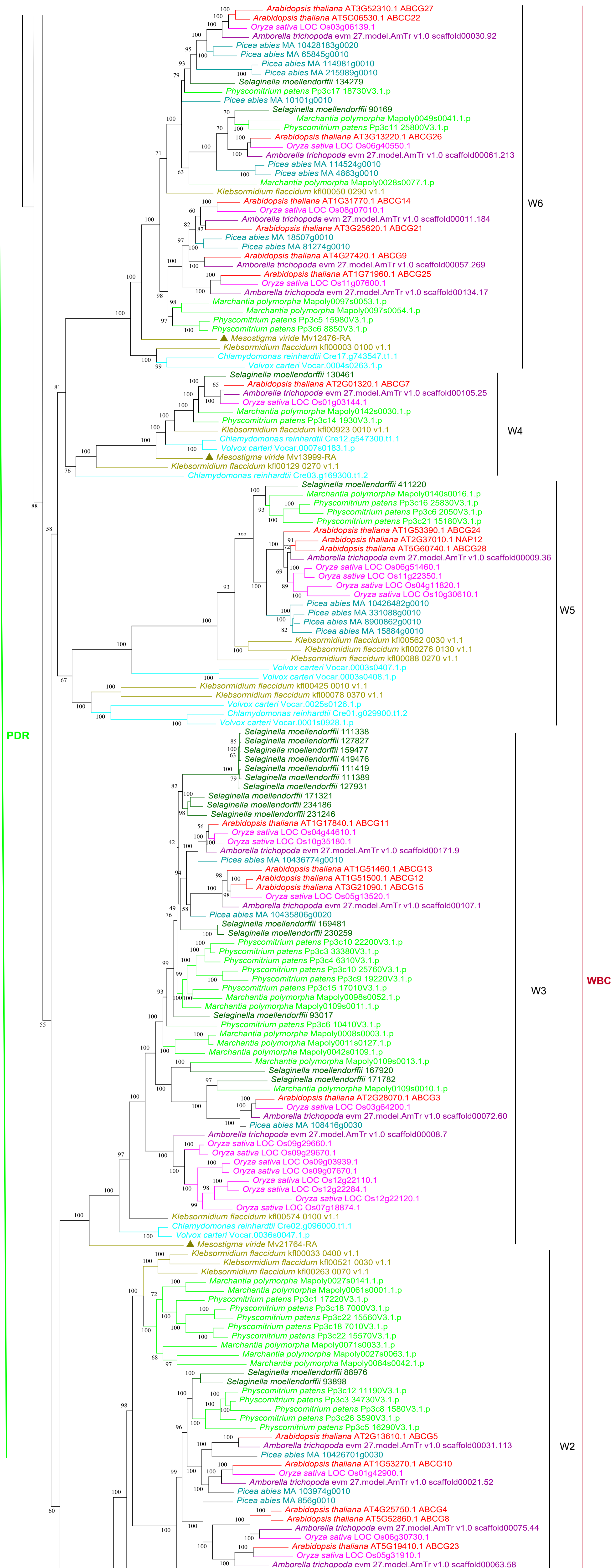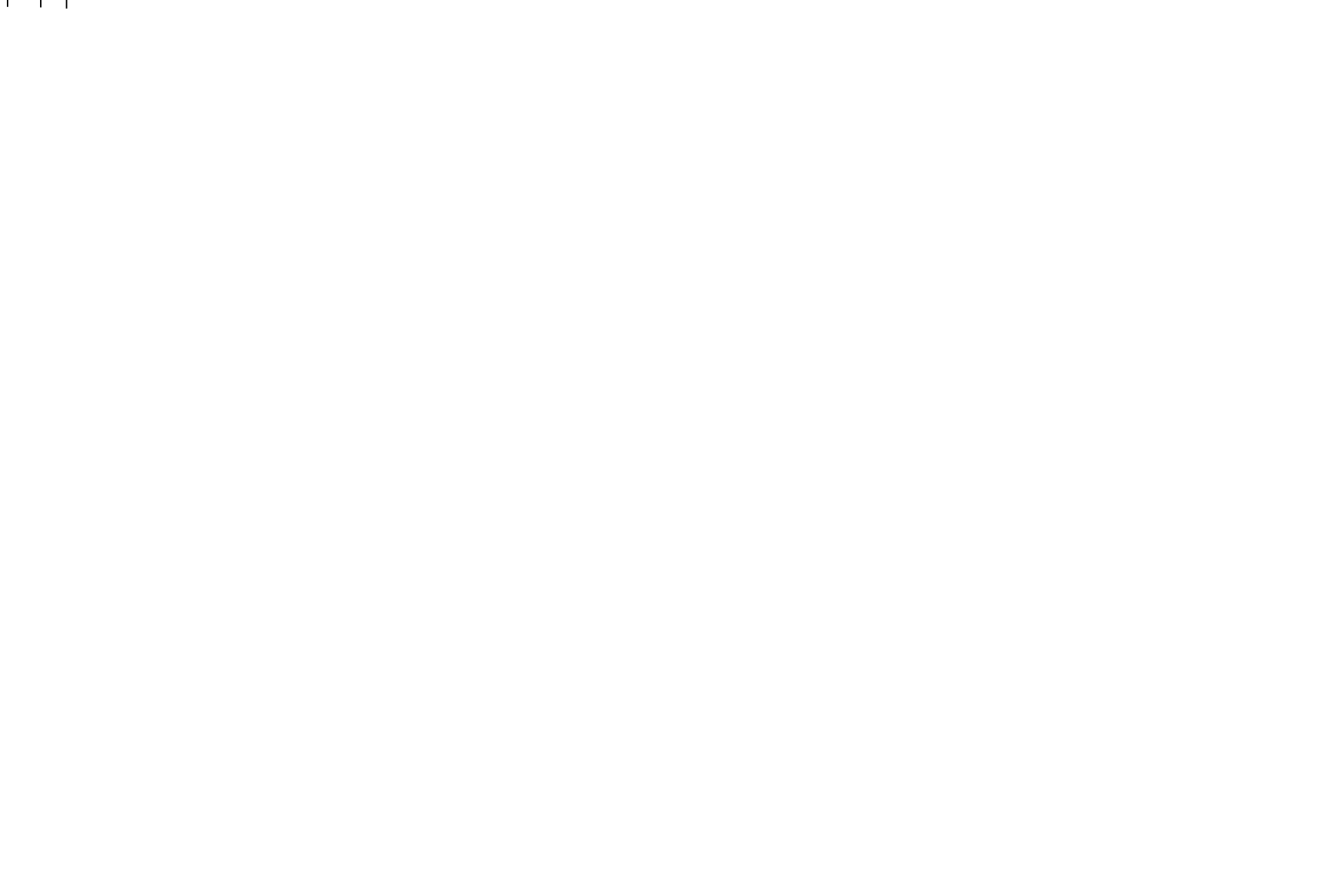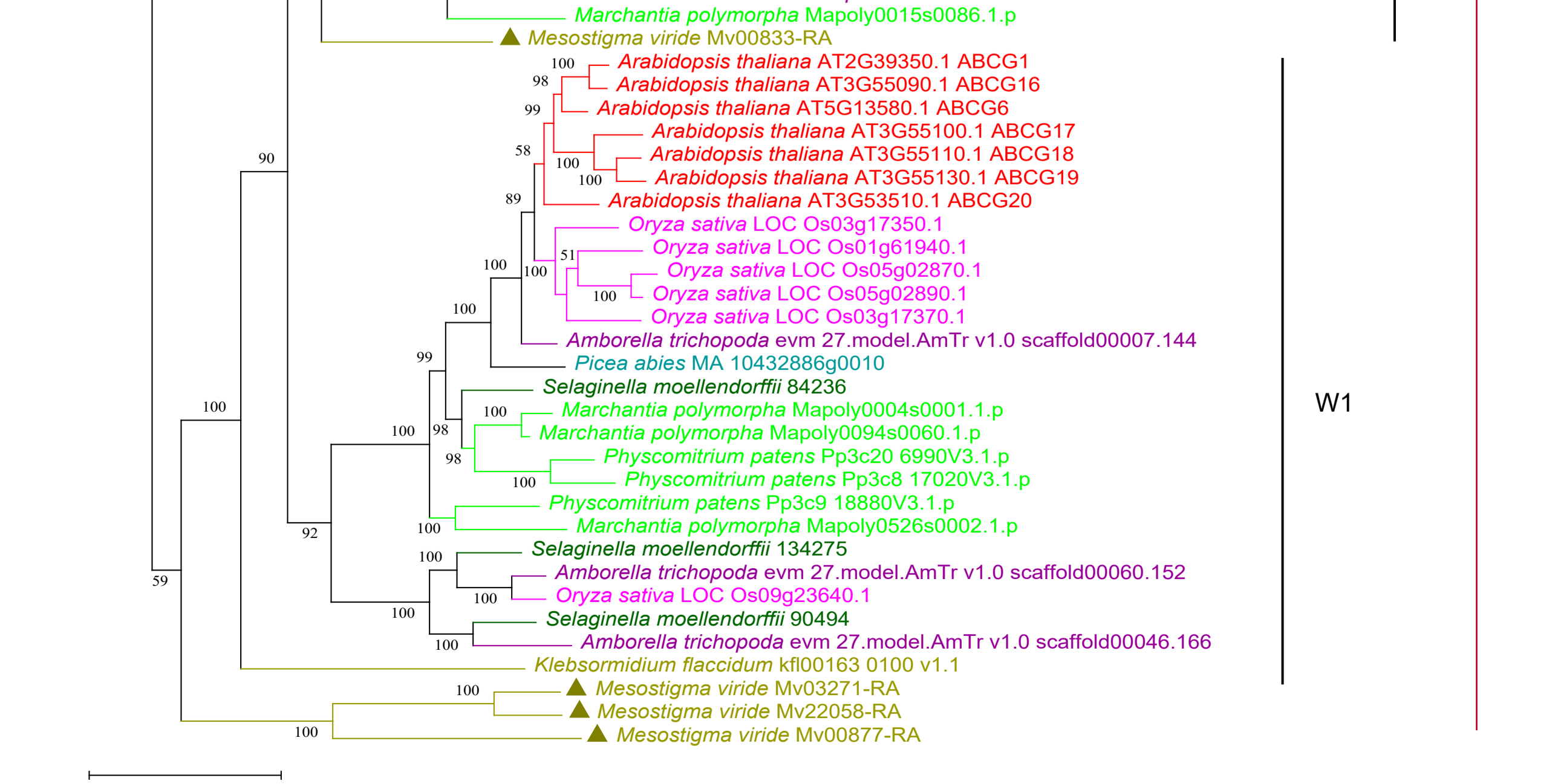

Supplement: Supplementary file 1 [file cimb-44-00112-s001.zip › Figure S8.pdf]

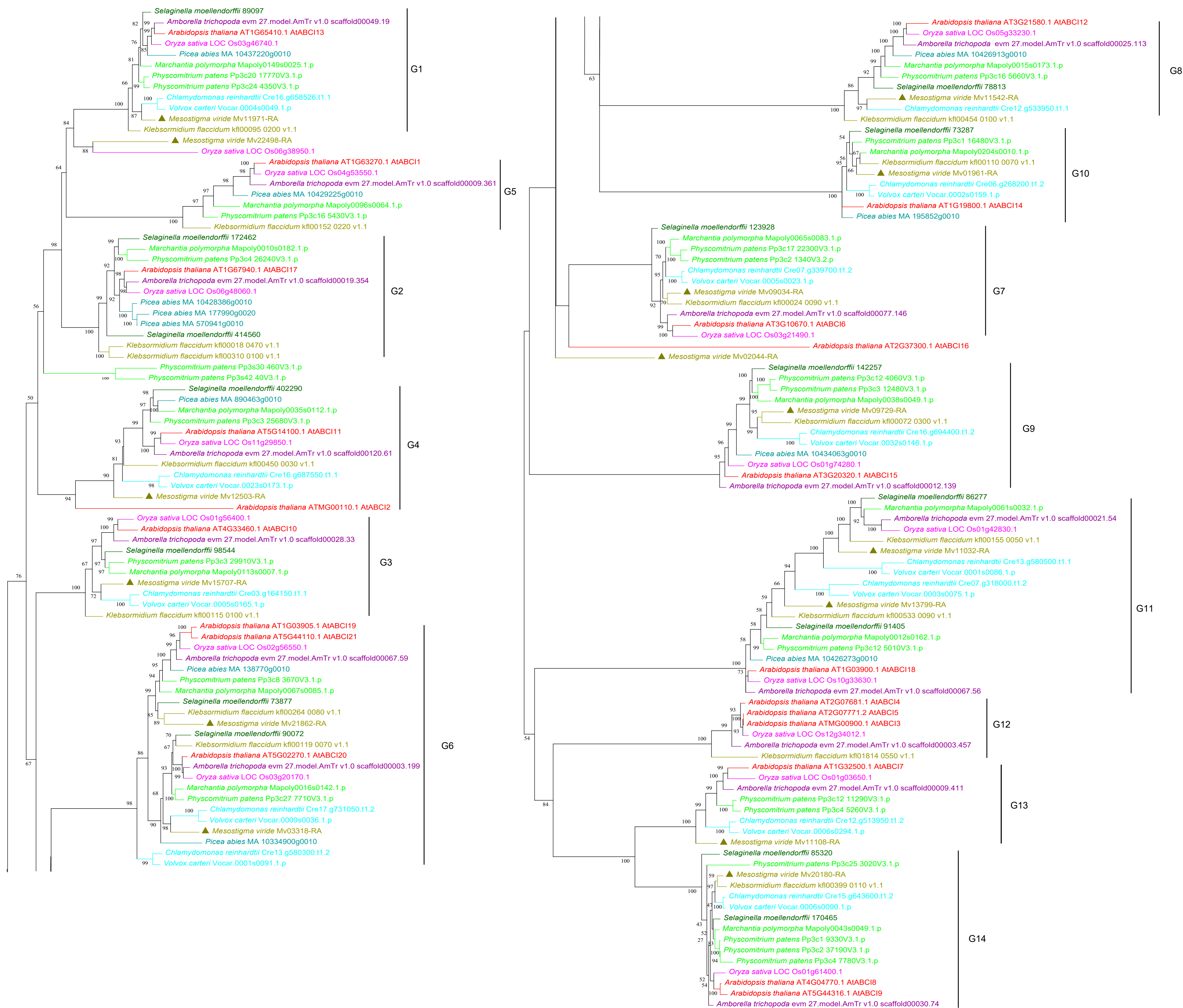

Supplement: Supplementary file 1 [file cimb-44-00112-s001.zip › Figure S9.pdf]
